# Prolactin promotes a partial recovery from the atrophy of both male and female gerbil prostates caused by castration

**DOI:** 10.1186/s12958-021-00777-2

**Published:** 2021-06-22

**Authors:** Marianna Zanatelli, Simone Jacovaci Colleta, Luiz Henrique Alves Guerra, Fernanda Cristina Alcântara Santos, Rejane Maira Góes, Patricia Simone Leite Vilamaior, Sebastião Roberto Taboga

**Affiliations:** 1 Department of Structural and Functional Biology, Institute of Biology, State University of Campinas – UNICAMP, SP Campinas, Brazil; 2 Laboratory of Microscopy and Microanalysis, Department of Biology, São Paulo State University – UNESP/IBILCE, Rua Cristóvão Colombo, 2265, Jardim Nazareth, SP 15054-000 São José do Rio Preto, Brasil; 3 Department of Morphology, Federal University of Goiás -UFG , GO Goiânia, Brazil

**Keywords:** Female prostate, Prostate, Gerbil, Castration, Prolactin, Prolactin receptor

## Abstract

**Background:**

The male and female prostates are controlled by steroid hormones, suffering important morphological and physiological changes after castration. Prolactin is involved in the regulation of the male prostate, having already been identified in the tissue, acting through its receptor PRLR. In the Mongolian gerbil, in addition to the male prostate, the female prostate is also well developed and active in its secretion processes. The aim of the present study was to evaluate the effects of exposure to exogenous prolactin in the prostate of both intact and castrated male and female gerbils in order to establish if prolactin administration can sustain prostate cell activity in conditions of sexual hormone deprivation.

**Methods:**

The morphological analyses were performed by biometric analysis, lesion histological analysis and morphometric-stereological aspects. In addition, immune-cytochemical tests were performed for prolactin and its receptor, as well as for the receptors of androgen and oestrogen and serum prolactin dosage. All data were submitted to ANOVA or Kruskal-Wallis tests for comparison between groups. P < 0.05 was considered to be statistically significant.

**Results:**

The results showed a strong influence of prolactin on the morphology of the prostate, with the development of important epithelial alterations, after only 3 days of administration, and an expressive epithelial cell discard process after 30 days of administration. Prolactin acts in synergy with testosterone in males and mainly with oestrogens in females, establishing different steroid hormonal receptor immunoreactivity according to sex. It was also demonstrated that prolactin can assist in the recovery from some atrophic effects caused in the gland after castration, without causing additional tissue damage.

**Conclusions:**

The prolactin and its receptor are involved in the maintenance of the homeostasis of male and female gerbils, and also cause distinct histological alterations after exogenous exposure for 3 and 30 days. The effects of prolactin are related to its joint action on androgens and oestrogens and it can also assist in the recovery from the atrophic effects of castration.

## Background

In recent years, in addition to the many studies on prostate cancer and other male prostatic diseases, the female prostate has been given much attention due to studies reporting that the gland can suffer pathological changes such as prostatitis, benign prostatic hyperplasia and malignant neoplasm [1–3], as well as giving rise to clinical problems such as para-urethral cystitis [4], urinary tract infections [5], female urethral syndrome [6] and urethral adenocarcinomas [7]. The human female prostate, historically known as Skene’s para-urethral gland, is a group of glands arranged in ductal structures situated alongside the urethra, on the anterior vaginal wall [8], while the human male prostate is a walnut-shaped gland situated at the base of the bladder [9].

The rodent *Meriones unguiculatus* (Mongolian gerbil) has been used by our research group as an excellent model for female prostate studies, since the gland in these animals is homologous to the human female prostate, while the male gerbil ventral prostate is highly frequent and physiologically developed [10–13]. The male gerbil presents a multi-lobulated prostate, whose histology and ultrastructure are also comparable to the human prostate, such as the smooth muscle layer around the acini and the cell types constituting the glandular epithelium [14, 15]. The epithelium is composed of two principal cell types – secretory cells and basal cells, and the stroma is composed of fibroblasts and smooth muscular cells [16]. The female prostate is histologically similar to the male prostate. The ducts and alveoli are composed of a cubic epithelium. The epithelium may have apical cilia, a female-only feature. Basal cells can be found among the secreting cells. Surrounding this epithelium are concentric layers of smooth muscle interspersed with fibroblasts that together constitute the muscle stroma [11, 17]. Although there is no clear homology between prostate lobes and human prostate zones, many studies involving the manipulation of hormones have been directed at the ventral lobe, probably because it is a large lobe, sensitive to androgens and to lesion development [18, 19].

There is intriguing evidence of the involvement of peptide hormones in the regulation of male prostatic tissue [20, 21]. These hormones might directly influence the expression of genes, interact with signal transduction routes of steroid hormones, and probably mediate some properties of steroid actions [22]. The peptide hormone prolactin (PRL) is secreted mainly by the pituitary gland and, to a lesser extent, by peripheral tissues, such as the breast, decidua, prostate, and the brain, and it is involved in a broad spectrum of physiological processes in vertebrates [23]. The principal stimulatory and inhibitory control of prolactin secretion is a hypothalamic hormone that inhibits the prolactin secretion the dopamine. Estrogen promote an increase of prolactin secretion as a result of diminish of dopamine[24].

In mammals, PRL is primarily involved with lactation and the reproductive process, the mammary gland being the major target for hormone action [24]. Circulating prolactin is also detected in males, but at lower levels than in females [25]. Over recent decades, studies in animal models have suggested that prolactin participates in the normal development, growth and function of the male prostate gland [20, 22]. The discovery that the human prostate expresses prolactin and prolactin receptors (PRLR) demonstrated that it might be a direct target of prolactin [26]. Besides, studies with rats demonstrated that the prostate is an extra-pituitary organ of prolactin production, and the hormone produced can act locally as a growth factor by the autocrine or paracrine route [22, 25].

Clinical and experimental studies have demonstrated the pleiotropic role of prolactin, stimulating cellular proliferation and secretory activity in the prostate, under normal or pathological conditions [27]. PRL has been associated with a number of different forms of cancer, among them human breast and prostate cancer [23, 25]. Besides, hyperprolactinemy is associated with amenorrhea, galactorrhea, pseudo-pregnancy and infertility in women [28]. Human patients with benign prostatic hyperplasia or prostate cancer have higher blood levels of PRL [23]. Hyperprolactinemia caused enlargement and inflammation of the lateral rat prostate [29]. In addition, estrogen seems to be essential for the inflammatory role of PRL in the prostate, since in aromatase-deficient mice study, high levels of PRL were not sufficient to cause inflammatory effects [30].

The initial step in PRL action is the binding to a specific membrane receptor, the PRLR, which belongs to the class 1 cytokine receptor superfamily [25, 30] and has been a therapeutic target in treating cancer [31]. Prolactin receptors have been reported in rodent male prostates [21, 25, 31, 32], indicating that the gland is responsive to the hormone.

Overall, androgens are required for the normal growth and functional activities of the male prostate, while oestrogen and progesterone are primarily responsible for the homeostasis of the female gland [10, 33–35]. Although it has been shown that the exogenous action of prolactin on the prostate of rats has no influence from androgens [36], studies over the years have demonstrated that prolactin can act in synergism with androgens and that oestrogens stimulate PRL secretion by the anterior pituitary [24, 37–39]. Although the castrated and hyperprolactinemia inducted rat have been used as in vivo model for study of the hormonal regulation of normal and pathological prostate development [29].There have still been no studies of the action of prolactin on the gerbil prostate, a highly sensitive experimental model for endocrine deregulation [40]. In this context, the aim of the present study was to evaluate the effect of prolactin exposure on the male and female gerbil prostates both under normal steroid hormone conditions and after castration, in order to establish if prolactin administration can sustain prostate cell activity in conditions of sexual hormone deprivation.

## Materials and methods

### Experimental design

Adult (90 days) gerbils (48 females and 48 males) were provided by the breeding centre of São Paulo State University (UNESP; São José do Rio Preto, SP). The experiment was performed in accordance with the requirements of the Ethics Committee of Experimental Animals of Sao Paulo State University (protocol number: 053/2011 CEUA). Animals were maintained in plastic cages under conventional conditions (25 °C, 40–70 % relative humidity, 12 light/12 dark), with water and rodent food *ad libitum*.

**Fig. 1:**
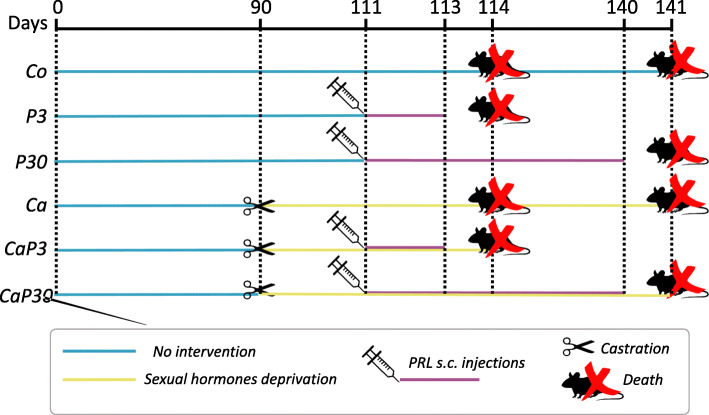
Illustrated diagram of the experimental design

All animals were euthanized by CO_2_ inhalation and decapitation after 3 or 30 days from the beginning of the administration of the drug. Females from the Co group were euthanized in the first pro-oestrus phase reached between 114 and 141 days of age, in order to prevent discrepancies in prostate histology relative to the oestrous cycle [13]. The pro-oestrus phase was determined by vaginal smear, according to Nishino and Totsucawa [32]. The males and females of the Co and Ca groups were euthanized at 114 or 141 days of age. During this period, histo-physiological and morphometric prostatic patterns remain practically unchanged, as had already been observed by previous studies of our research group [15, 33, 34], making it possible to use the same animals in comparison with 3-day and 30-day treated groups. All animal-handling procedures were carried out during the morning (between 8:00 and 10:00 a.m.). The prostate glands were dissected, together with the urethra (prostatic complex), and were weighed and fixed. The pituitary glands were also weighed for biometric analysis (Fig. 1).

### Histochemistry

For light microscopy, three prostatic fragments per group were fixed for 24 h in Karnovsky fixative (0.1 M Sörensen phosphate buffer, pH 7.4, containing 5 % paraformaldehyde and 2.5 % glutaraldehyde) and embedded in historesin (Leica Historesin Embedding Kit™, Nussloch, Germany), and the five others in 4 % paraformaldehyde in phosphate-buffered saline and embedded in paraffin (Histosec™; Merck, Darmstadt, Germany). The historesin-embedded fragments were cut into sections of 3 μm and submitted to staining by hematoxylin–eosin (H&E) and Periodic Acid and Schiff (PAS). The tissue sections were analysed in a Olympus BX60 light microscope (Olympus, Tokio, Japan) and microscopic fields were digitalized using the Image-Pro-Plus software version 4.5 for Windows (Media Cybernetics, Inc., Bethesda, USA).

### Stereological, morphometric and karyometric analysis

For all the measurements, H&E stained slides were used. The stereological analyses were carried out using Weibel’s M130 multipoint test system [35] to calculate the relative frequency of each component of prostatic tissue (epithelium, lumen, smooth muscular layer and non-muscular stroma), as described by Huttunen et al. [36]. For this, 12 random fields were captured, which covered the entire prostate extension of each animal, totalling 36 fields per experimental group. To determine epithelium height (µm) and smooth muscle layer thickness (µm), besides the nuclear area (µm^2^) and perimeter (µm) of secretory cells, morphometric and karyometric analysis was performed, respectively. For these analyses, 200 measurements were obtained for each experimental group.

### Histopathological analysis

Prostate lesions and inflammation were assessed by incidence and multiplicity. Histological sections stained with H&E were evaluated under light microscopy at 400x magnification using an Olympus BX-60 microscope. The evaluated histological sections were evenly spaced along the blocks used for each animal (*n* = 8). Diagnosis of morphological changes and inflammatory disorders was performed according to Shappell et al. [19], Dema et al. [37] and Gonçalves et al. [38]. For the quantification of prostate lesions, we considered intraepithelial neoplasms (PINs) and cribiform intraepithelial neoplasms (PINCs), characterized by regions with stratification of cells with evident nucleoli, polymorphism and nuclear enlargement. The quantification of inflammation was undertaken by counting inflammatory foci, characterized by the presence of intraluminal or pronounced epiductal cells of the immune system such as lymphocytes, plasma cells and neutrophils.

For prostate lesions and inflammation, the multiplicity was estimated by counting foci of disorders per section and the results expressed in mean ± SD per experimental group, and the incidence was estimated by the number of animals exhibiting each type of disorder and expressed as a percentage.

### Serum prolactin concentration

Eight animals per group were used for the determination of plasma prolactin. Blood samples were collected from the trunk of decapitated gerbils into test tubes with 4mL separation gel. Plasma was separated by centrifugation (3000 rpm for 15 min at 4 °C) and stored at − 80 °C for posterior analysis. Prolactin plasma was assayed by the double-antibody radio-immunoassay (RIA) method, with specific kits provided by the National Hormone and Peptide Program (Harbor-UCLA, USA). The antiserum and reference preparations were anti-rat PRL-S9 and PRL-RP3, respectively. The lower limit of detection was 0.19 ng/mL, and the intra-assay coefficient of variation was 4 %.

### Immunocytochemistry

For the analysis, paraffin sections were deparaffinized, and rehydrated through graded alcohols and distilled water. Antigen retrieval was performed in citrate buffer (citric acid monohydrate, 0.21 %, pH 6.0), at 98 °C for 20–50 min. The blockade of endogenous peroxidases was achieved by covering the slides with H_2_O_2_ (3 % in methanol) for 20 min, and the blockade of non-specific protein–protein interactions was achieved by incubating sections in 5 % powdered skim milk solution diluted in washed buffer for 30–60 min. Sections were incubated with the following primary antibodies diluted in 1 % bovine serum albumin (BSA) in washed buffer at 4 °C overnight: anti-androgen receptor (AR, rabbit polyclonal, clone N-20, dilution 1:50, Santa Cruz Biotechnology), anti-oestrogen receptor alpha (ERα, rabbit polyclonal, clone MC-20, dilution 1:100, Santa Cruz Biotechnology), anti-oestrogen receptor beta (ERβ, rabbit polyclonal, clone H-150, dilution 1:50, Santa Cruz Biotechnology), anti-prolactin receptor (PRLR, mouse monoclonal, clone U5, dilution 1:100, Abcam), anti-prolactin (PRL, goat polyclonal, clone C-17, dilution 1:75, Santa Cruz Biotechnology). The use of these antibodies was appropriate in gerbils because of the high level of sequence conservation of these proteins in mammals [24, 39]. Sections were then incubated with NovoLink Max Polymer detection system (Leica), revealed with diaminobenzidine (DAB; Sigma) and counterstained with Harris Hematoxylin. As a negative control, the primary antibodies were replaced with BSA 1 %. Immunostaining was assessed using an Olympus BX-60 light microscope and counts were performed using Image-Pro Plus software, Version 4.5 for Windows (Media Cybernetics). The PRLR, PRL, Erα, Erβ and AR indices were expressed as percentages of positive cells from the total cells, counted in 30 microscopic fields randomly selected from each experimental group. A minimum of 1000 cells were counted, and then the percentage of immunoreactivity was calculated as the number of positive cells divided by the total number of cells. The intensity of staining was not taken into consideration, and all cells with positively stained nuclei or cytoplasm (depending on the location of each marker) were considered to be positive. All images and quantitative measurements were performed by the investigators blinded to both the animal identity and experimental condition.

### Statistical analyses

All quantitative data were subjected to statistical analysis with Prism 6.01 software (GraphPad Software, Inc. CA, USA). All data were assessed for normality and then submitted either to a ANOVA test with a Tukey post-test (for parametric data), or to a Kruskal-Wallis test with a Dunn post-test (for non-parametric data). The results were presented in terms of the mean ± standard deviation and p values ≤ 0.05 were considered to be statistically significant.

## Results

### PRL affected relative prostatic complex weight and caused changes in other biometric parameters

**Fig. 2:**
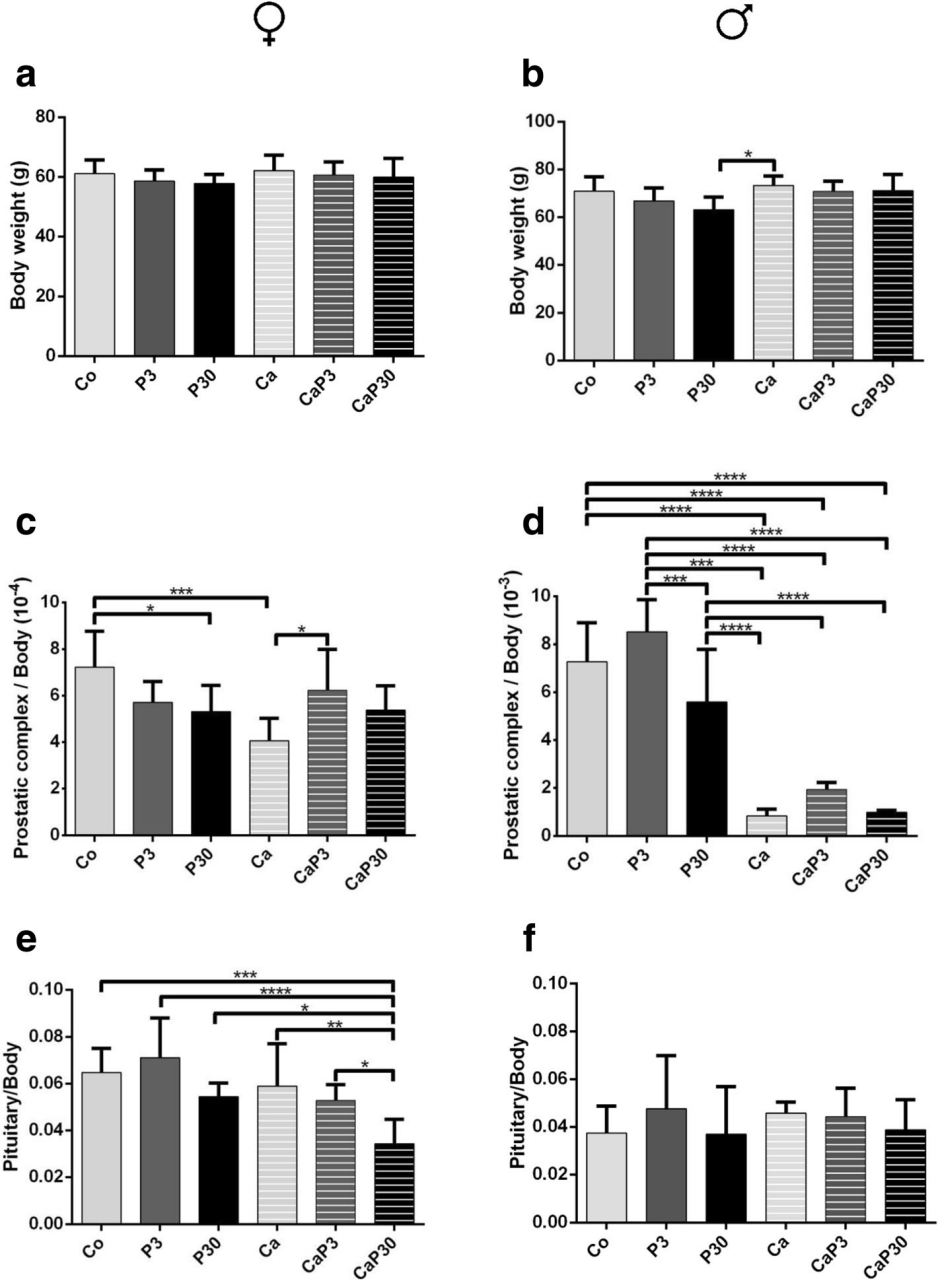
Biometric data of female (left graphics) and male (right graphics) gerbils from different experimental groups. Values expressed as mean ± SD. **a** Body weight. **b** Prostate complex relative weight. **c** In left ovaries, in right testicles. **d** Adrenals weight. **e** Pituitary weight. Different letters represent statistically significant differences between the experimental groups, p ≤ 0.05. Statistical analysis based on ANOVA A and Tukey’s tests

Body weight did not alter in the female group. In males, long administration of prolactin (P30) caused a reduction in body weight when compared with the Ca group (Figs. 1 and 2a, b).

In females, castration (Ca), as well as long treatment with prolactin (P30), caused a decrease in prostate complex relative weight when compared to the control group (Co). Short treatment with prolactin in castrated animals (CaP3), on the other hand, showed weight recovery compared to the Ca and P30 groups (Fig. 2c).

In males, castration (Ca) caused a reduction in relative prostatic complex weight compared to uncastrated animals (Co, P3 and P30). The administration of prolactin in the castrated animals did not interfere in the prostatic relative weight compared to that of the castrated group (Ca) (Fig. 2d).

In females, the CaP30d group showed a decreased in pituitary relative weight in females compared to other groups, while in males no significant differences were found between the groups (Fig. 2e, f).

### PRL caused significant morphological changes in the prostate and recovery from the atrophic effects of castration

**Fig. 3:**
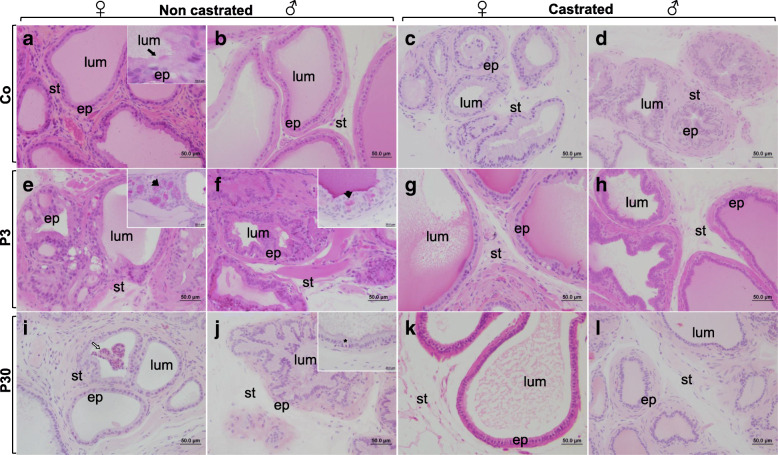
Histological aspects of the gerbil female and male ventral prostates from different experimental groups stained with H&E and Periodic acid and Schiff (E and F inset). **a** and **b**- Prostate from the Co group showing a normal morphologic pattern of the gland. **c** and **d**- Castrated gland from the Ca group with intense regression, observed by the decrease in lumens area and the epithelium tortuosity **e** and **f**- P3d group showed expressive histological alterations, note the cribriform architecture throughout the gland, in details inset note the microacini formed with secretion. **g** and **h-** CaP3 group recovery of atrophic effects of castration, as acini more voluminous and rounded, epithelium healthier, sml more organized compared with the castrated animals. **i** and **j-** The morphological atypia in the epithelium were substantially reduced when compared with P3, observe epithelial cells debris into the lumen and the inset details in **j** a epithelial cell show a clear halo around the pyknotic nucleus. **k** and **l-** CaP30 group presents recovery of atrophic effects of castration

When compared to the Co group (Fig. 3a, b), the P3 group (Fig. 3e, f) showed expressive histological alterations in both the male and female prostates. These alterations included multiple regions with morphological atypia in the epithelium, mostly resulting in a cribriform architecture throughout the gland and the presence of inflammatory cells in the subepithelial and stromal regions. In addition to these disorders, only uncastrated groups showed an incidence of inflammation, with the exception of the CaP3 group for males (Table 1). In acini with lesions, in females, ciliated cells were observed grouped and not isolated as is commonly observed in control females (Fig. 3a inset). In general, there was an apparent reduction in glycoprotein secretion production, but this secretion was also observed within the microacini formed in altered epithelium (Fig. 3e, f inset). On the other hand, in females and males from the P30 (Fig. 3i, j and Table 1) groups, the morphological atypia in the epithelium were substantially reduced when compared with P3. Furthermore, some epithelial cells showed a clear halo around the pyknotic nucleus (Fig. 3j inset). Many acini showed epithelial cell debris in the lumen (Fig. 3i,j).

**Table 1:** Incidence and Multiplicity of inflammations and morphological alterations observed in prostates

		**Co**	**P3**	**P30**	**Ca**	**CaP3**	**CaP30**
		**Inflammation**					
**Incidence (%)**	**♀**	12.5	25.0	25.0	0	0	0
	**♂**	25.0	50.0	25.0	0	13.0	0
**Multiplicity (mean ± SD)**	**♀**	0.037 ± 0.1	0.061 ± 0.1	0.082 ± 0.1	0	0	0
	**♂**	0.227 ± 0.4	0.435 ± 0.6	0.220 ± 0.4	0	0.041 ± 0.1	0
		**Morphological alterations**					
**Incidence (%)**	**♀**	62.5	75.0	12.5	0	62.5	0
	**♂**	12.5	50.0	12.5	0	0	0
**Multiplicity (mean ± SD)**	**♀**	0.310 ± 0.3^a,b^	1.333 ± 1.7^a^	0.082 ± 0.2^a,b^	0^b^	0.405 ± 0.5^a,b^	0^b^
	**♂**	0.020 ± 0.05^a,b^	1.040 ± 1.8^a^	0.020 ± 0.05^a,b^	0^b^	0^b^	0^b^

Different letters represent statistically significant differences between the experimental groups, *p* ≤ 0.05

After castration, both female and male prostates suffered acinar regression, observed by the decrease in lumens and the epithelium tortuosity (Fig. 3c, d). In general, animals from the CaP3 (Fig. 3 g, h) and CaP30 (Fig. 3k, l) groups showed the same recovery from atrophic effects, such as more voluminous and rounded acini, healthier epithelium, and more organized sml.

#### Morphometric-stereological and kariometrical analysis

The results obtained with the stereological, morphometric and kariometric analyses are presented in Table 2.

**Table 2:** Stereological, Morphometric and Kariometric data from different experimental groups (mean ± SD)

**Parameters**		**Co**	**P3**	**P30**	**Ca**	**CaP3**	**CaP30**
***Stereological (%)***							
Epithelium	**♀**	28.70 ± 7.1 a	29.08 ± 10.3 a	24.22 ± 10.2 a,b	25.60 ± 10.4 a,b	20.92 ± 6.4 b,c	16.30 ± 4.6 c
	**♂**	29.29 ± 7.5	30.75 ± 10.8	25.40 ± 10.1	27.25 ± 8.5	25.58 ± 5.6	28.51 ± 9.0
Lumen	**♀**	34.61 ± 19.2 a	28.96 ± 17.6 a	34.95 ± 16.0 a	28.41 ± 13.4 a	25.69 ± 8.3 a	14.32 ± 6.7 b
	**♂**	36.62 ± 14.4 a,b	34.33 ± 17.4 a,b	44.58 ± 14.1 b	24.30 ± 12.6 c	25.81 ± 10.3 a,c	18.12 ± 11.7 c
SML	**♀**	16.76 ± 6.4 a	18.27 ± 3.2 a,b	17.76 ± 5.4 a,b	21.53 ± 6.4 b,c	25.20 ± 9.3 c	26.9 ± 11.0 c
	**♂**	24.44 ± 6.6 a,b	21.72 ± 7.2 b	19.37 ± 5.9 b	29.25 ± 5.6 a,c	32.76 ± 7.0 c,d	37.29 ± 8.5 d
Non-muscular Stroma	**♀**	20.50 ± 9.6 a	23.77 ± 10.0 a,b	23.24 ± 11.5 a,b	24.6 ± 14.0 a,b	28.43 ± 8.3 b,c	43.03 ± 16.9 c
	**♂**	9.82 ± 6.0 a	13.39 ± 8.9 a,b	10.87 ± 5.8 a,c	19.40 ± 9.9 b	16.01 ± 9.6 a,b	16.22 ± 9.2 b,c
***Morphometric (µm)***							
Epithelium height	**♀**	13.10 ± 3.1 a	15.81 ± 5.0 b,c	17.79 ± 6.3 c	9.47 ± 4.3 d	10.77 ± 3.4 d	15.44 ± 4.9 b
	**♂**	19.66 ± 3.9 a	16.19 ± 4.5 b	14.07 ± 3.9 c	31.63 ± 10.4 d	14.64 ± 4.3 b,c	13.52 ± 5.3 b,c
SML thickness	**♀**	8.56 ± 3.9 a	10.27 ± 3.0 b	16.13 ± 5.4 c	6.32 ± 2.6 d	9.68 ± 3.1 b	18.00 ± 6.3 c
	**♂**	15.23 ± 4.2 a	10.81 ± 3.9 b	12.10 ± 3.9 b	38.75 ± 12.9 c	14.56 ± 3.8 a	16.15 ± 5.0 a
***Kariometric***							
Nuclear area (µm^2^)	**♀**	12.89 ± 3.0 a	14.75 ± 3.8 a	30.36 ± 5.7 b	8.84 ± 2.4 c	13.12 ± 2.5 a	27.56 ± 5.4 b
	**♂**	31.09 ± 5.2 a	26.37 ± 5.3 b	24.35 ± 5.7 c	22.36 ± 5.7 d	27.07 ± 5.1 b	24.20 ± 5.4 c
Nuclear perimeter (µm)	**♀**	15.63 ± 2.3 a,b	15.64 ± 2.0 b	21.32 ± 1.9 c	12.46 ± 1.7 d	14.67 ± 1.6 a	20.73 ± 2.3 c
	**♂**	24.92 ± 3.1 a	20.45 ± 2.7 b	20.38 ± 2.7 b	19.39 ± 2.6 c	21.05 ± 2.2 b	20.11 ± 3.0 b,c

Different letters represent statistically significant differences between the experimental groups, *p* ≤ 0.05

### PRL caused opposite effects in the epithelial height and sml thickness of the male and female prostate

The castration of females promoted a decrease in epithelial height and sml thickness (Co vs. Ca). The administration of PRL promoted increased epithelial height and sml thickness (P3 and P30 vs. CO). The same effects were observed in the sml thickness of castrated groups (Ca vs. CaP3 and CaP30). However, the epithelial height only increased in ovari-ectomized CaP30 groups.

In males, surgical castration caused an increase in epithelial height and sml thickness (Co vs. Ca). The administration of PRL promoted a decrease in sml thickness in uncastrated (Co vs. P3 and P30) and in castrated groups (Ca vs. CaP3 and CaP30). With regard to epithelial height, PRL caused a decrease in the P3 and P30 groups in relation to the CO group; this effect was more marked after 30 days of treatment. In the castrated group, PRL promoted a similar decrease in epithelial height in both the CaP3 and CaP30 groups compared to the Ca group.

### PRL caused changes in the nuclear area and perimeter

In females, 30 days of PPL administration caused an increase in the nuclear area and perimeter in uncastrated and castrated groups. The castrated animals (Ca group) had a decrease in the nuclear area and perimeter when compared with uncastrated animals (Co group).

In uncastrated males, the nuclear area had a decrease in the P3 and P30 groups when compared with the Co group; this effect was more marked after 30 days of treatment. On the other hand, in castrated animals, the nuclear area showed an increase. With regard to the nuclear perimeter, the P3 and P30 groups showed a decrease in perimeter in relation to the Co group. Among the castrated animals, only the CaP3 group showed an increase in nuclear perimeter.

Both Ca and Co animals had a decrease in nuclear area and perimeter.

### Stereological analysis

In uncastrated females, the frequency of epithelium, stroma, lumen and sml did not change when treated with prolactin. In castrated animals, the epithelium and lumen showed a frequency decrease in the CaP30 group, while the stroma presented a frequency increase, and the CaP3 and CaP30 groups showed greater sml frequency (Table 3).

**Table 3:** Prolactin serum concentrations (ng/mL) in gerbils from different experimental groups

**Sex**		**Groups**				
	**Co**	**PRL 3d**	**PRL 30d**	**Ca**	**CaPRL 3d**	**CaPRL 30d**
Female	6.57 ± 1.1 a	4.64 ± 0.7 b	4.37 ± 0.8 b,c	4.72 ± 0.5 b	4.45 ± 0.5 b	4.59 ± 1.0 c
Male	3.75 ± 0.6 a	4.17 ± 0.6 a,c	3.83 ± 0.5 b,c	5.12 ± 0.5 b	4.86 ± 0.8 b	4.66 ± 0.5 b,c

Different letters represent statistically significant differences between the experimental groups, *p* ≤ 0.05

The comparison of uncastrated animals with their corresponding castrated group showed a decrease in CaP3 and CaP30 epithelial frequency; however, the sml compartment had an increase in frequency. The CaP30 group showed an increase in stroma frequency and a decrease in lumen (Table 3).

In males, the epithelial compartment remained unaltered in all the groups. A comparison between uncastrated animals and their corresponding castrated group showed an increase in stroma Ca vs. Co. Castration increased the size of the SML compartment and this effect was enhanced with prolactin treatment. The comparison between uncastrated animals and their corresponding castrated group showed an increase in SML in both CaP3 and CaP30 groups. With regard to lumen, the castrated groups had a diminution in frequency when compared to uncastrated groups (Table 3).

#### PRLR and PRL presented the same immunostaining pattern

**Fig. 4:**
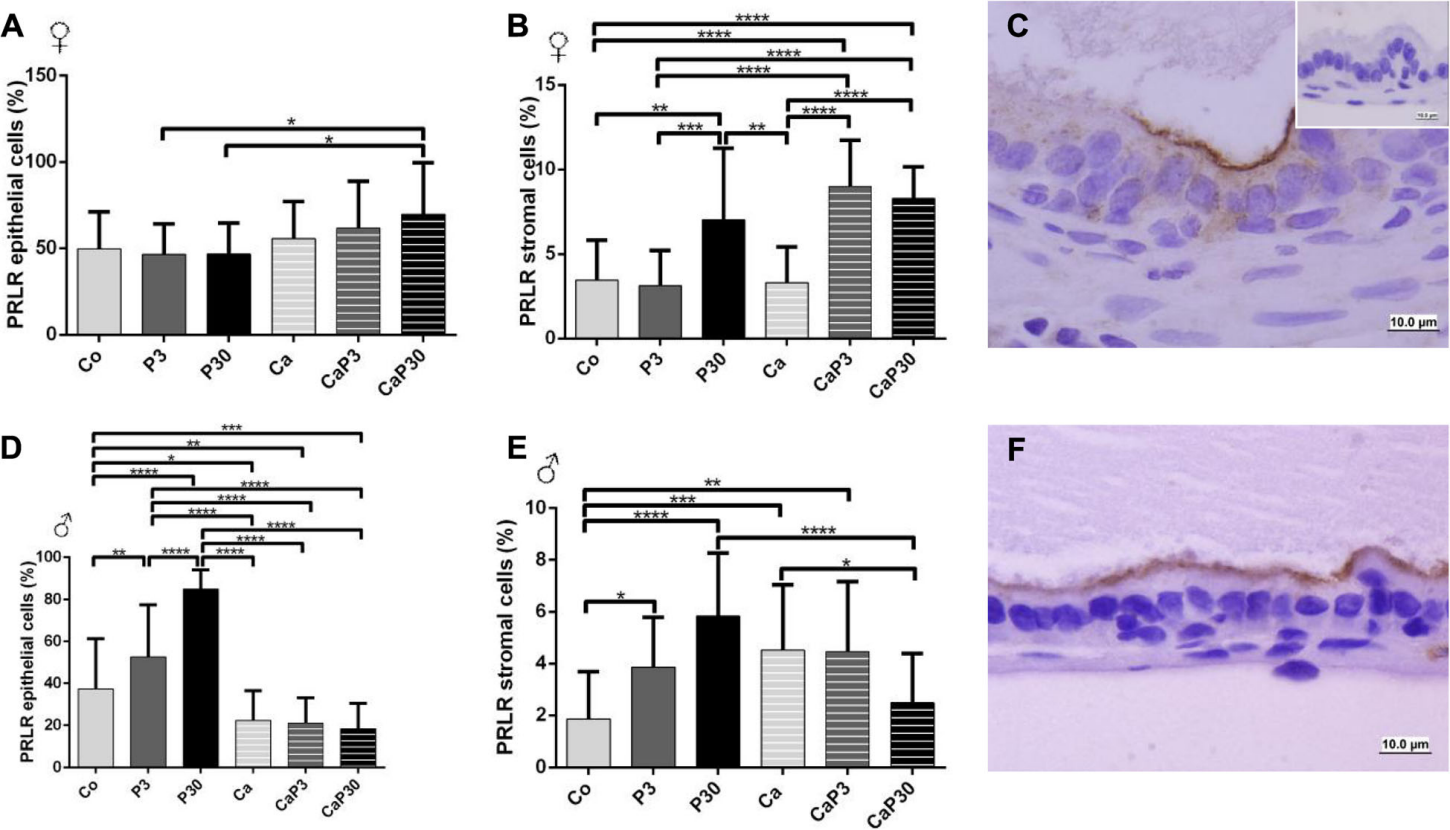
Graphics obtained by epithelial and stromal cell immunostaining count for PRLR and their respective immunocytochemistry photomicrographs. Counter-stained with Harris hematoxylin. Black arrows indicate immunostaining of epithelial cell, white arrows indicate immunostaining of stromal cells. Inset refer to negative controls for the technique. Values are expressed as mean in percentage ± SD. Different letters represent statistically significant differences between the experimental groups, p ≤ 0.05. Statistical analysis based on ANOVA A and Tukey’s tests

**Fig. 5:**
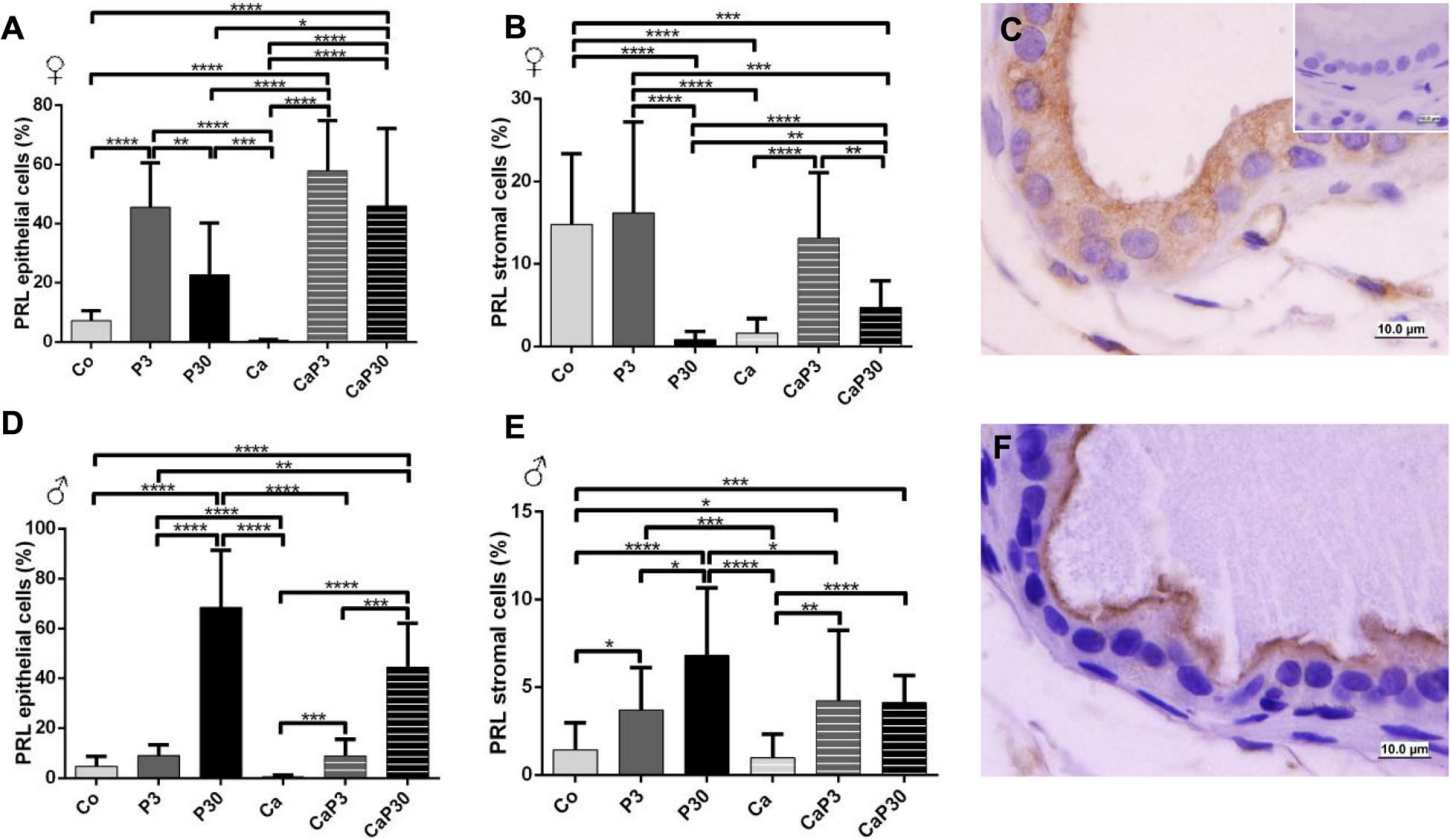
Graphics obtained by epithelial and stromal cell immunostaining count for PRL and their respective immunocytochemistry photomicrographs. Counter-stained with Harris hematoxylin. Black arrows indicate immunostaining of epithelial cell, white arrows indicate immunostaining of stromal cell. Inset refer to negative controls for the technique. Values are expressed as mean in percentage ± SD. Different letters represent statistically significant differences between the experimental groups, p ≤ 0.05. Statistical analysis based on ANOVA A and Tukey’s tests

The presence of the prolactin receptor (PRLR) and the soluble prolactin (PRL) in male and female gerbil prostates was studied by immunocytochemistry. Gerbil pituitaries were used as positive control tissues. PRLR and PRL both presented the same immunostaining pattern (Figs. 4c and f and 5c and f). Both were mainly located on the apical surfaces of the secretory epithelial cells of prostatic acini, but also appeared scattered throughout the cytoplasm. The nucleus of the cells remained unstained. A low number of stromal cells showed positive staining of the cytoplasm. In some acini, an intensive positive reaction was localized to single epithelial cells scattered within the cytoplasm.

In females, immunostained cell counts data showed that there was no alteration in immunostained PRLR in castrated animals (Ca) when compared to control (Co), but decreased in PRL for stromal cells. When prolactin was administered to castrated females, it increased PRL in the epithelial cells and stromal cells of the CaP3 group, but PRLR was only increased in stromal cells of the CaP3 group (Fig. 4a, b).

In the uncastrated groups, the P3 group showed an increase in PRL epithelial cells when compared with the Co and P30 groups. The P30 group also showed a decrease in PRL stromal cells and an increase in PRLR stromal cells in comparison with the CO and P3 groups (Fig. 5a, b).

In males, castration decreased PRLR immunostaining in epithelial cells and increased it in stromal cells (CO vs. Ca). In uncastrated males, administration of exogenous prolactin increased PRLR immunostaining in both epithelial and stromal cells, and this effect was more pronounced after 30 days of treatment (Fig. 4d, e). Prolactin increased the PRL immunostained cells in stroma and, after 30 days of treatment, in epithelial cells as well (Fig. 5e). In castrated males, administration of prolactin only decreased PRLR in stromal cells in the CaP30 group. However, PRL immunostaining increased in both epithelial and stromal cells (Fig. 4d, e).

#### Treatment with prolactin altered ERα and ERβ immunostaining

**Fig. 6:**
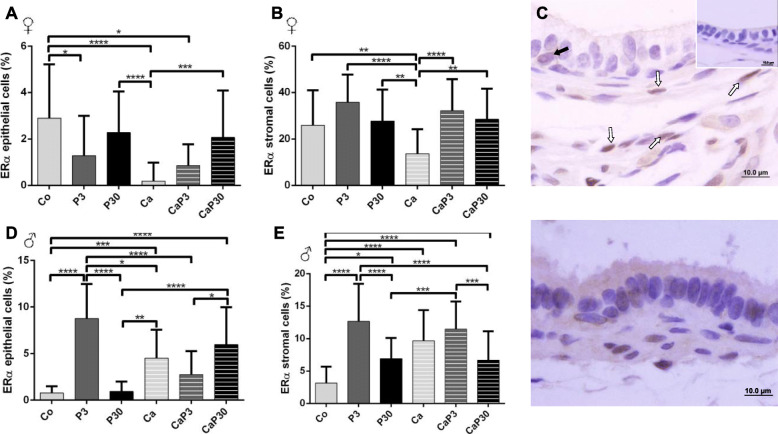
Graphics obtained by epithelial and stromal cell immunostaining count for ERα and their respective immunocytochemistry photomicrographs. Counter-stained with Harris hematoxylin. Black arrows indicate immunostaining of epithelial cell, white arrows indicate immunostaining of stromal cell. Inset refer to negative controls for the technique. Values are expressed as mean in percentage ± SD. Different letters represent statistically significant differences between the experimental groups, p ≤ 0.05. Statistical analysis based on ANOVA A and Tukey’s tests

Castration reduced ERα immunostaining in epithelial and stromal cells of the female prostate (Co vs. Ca). Treatment with prolactin for 3 days in uncastrated females decreased ERα immunostaining in epithelial cells. In castrated females, there was an increased ERα count in stromal cells in CaP3 and CaP30 group and in epithelial cells only in CaP30 group (Fig. 6a, b).

In males, castration increased ERα immunostaining in epithelial and stromal cells of the prostate (Co vs. Ca). The administration of exogenous prolactin in uncastrated males increased ERα immunostained cells in stroma in both groups (P3 and P30), but epithelial cells only increased in the P3 group. In castrated males, the CaP3 group showed an increase in immunostained cells in the epithelium and a decrease in the stroma in comparison with CaP30 (Fig. 6d, e).

**Fig. 7:**
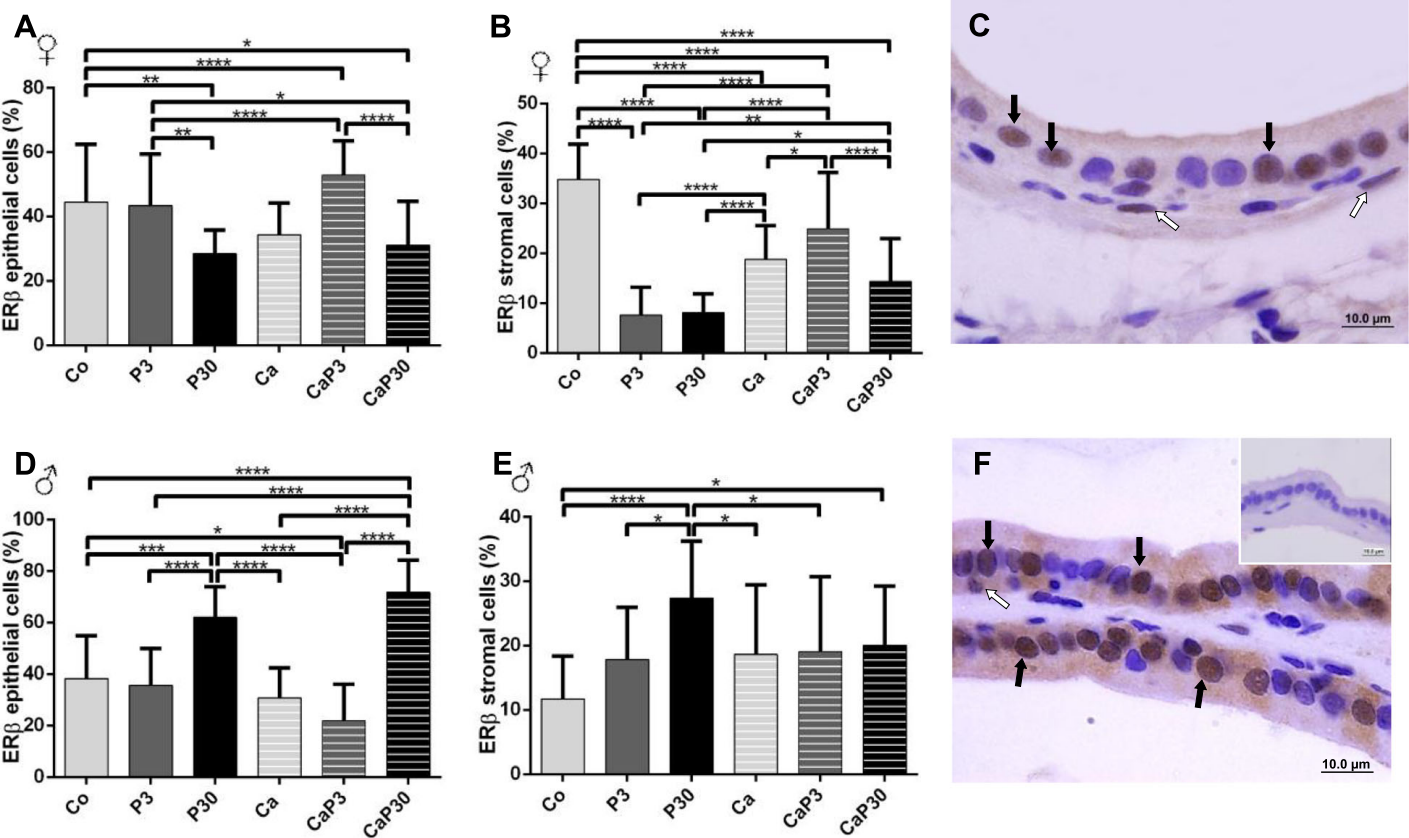
Graphics obtained by epithelial and stromal cell immunostaining count for ERβ and their respective immunocytochemistry photomicrographs. Counter-stained with Harris hematoxylin. Black arrows indicate immunostaining of epithelial cell, white arrows indicate immunostaining of stromal cell. Inset refer to negative controls for the technique. Values are expressed as mean in percentage ± SD. Different letters represent statistically significant differences between the experimental groups, p ≤ 0.05. Statistical analysis based on ANOVA A and Tukey’s tests

Castration decreased ERβ stromal cell immunostaining for females. Exogenous prolactin administered in uncastrated females decreased ERβ immunostaining for stromal cells in both groups and for epithelial cells when the animals were treated for 30 days. When administered for 3 days in castrated animals, exogenous prolactin increased ERβ immunostaining in both epithelial and stromal cells (Fig. 7a, b).

In males, castration did not alter male prostatic cell ERβ immunostaining. When administered for 30 days, exogenous prolactin increased ERβ epithelial immunostaining in both intact and castrated males (P30 and CaP30) and the P30 group showed an increase in immunostained stromal cells (Fig. 7d, e).

#### Castration did not alter AR immunostaining

**Fig. 8:**
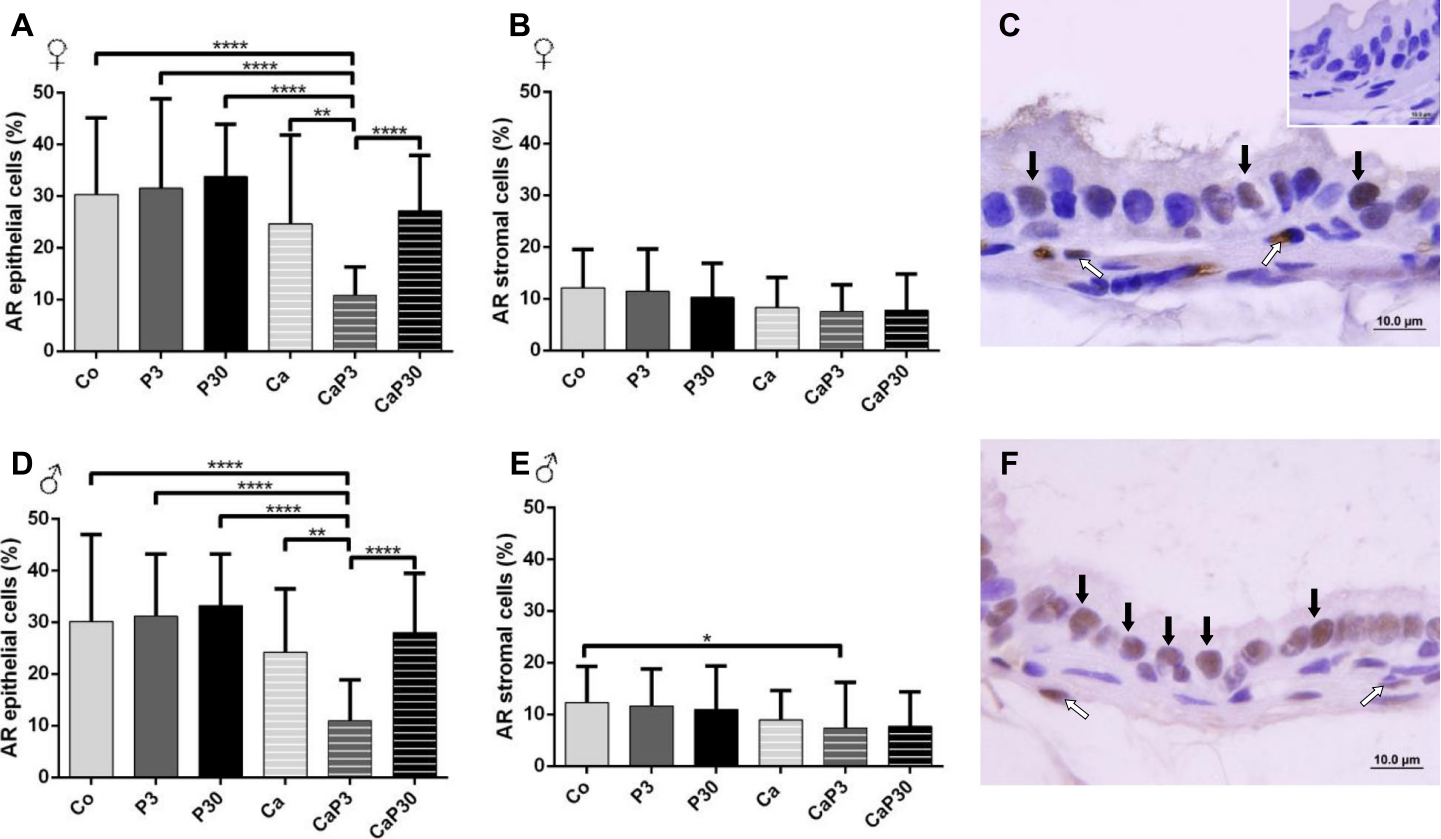
Graphics obtained by epithelial and stromal cell immunostaining count for AR and their respective immunocytochemistry photomicrographs. Counter-stained with Harris hematoxylin. Black arrows indicate immunostaining of epithelial cell, white arrows indicate immunostaining of stromal cell. Inset refer to negative controls for the technique. Values are expressed as mean in percentage ± SD. Different letters represent statistically significant differences between the experimental groups, *p* ≤ 0.05. Statistical analysis based on ANOVA A and Tukey’s tests

Castration did not alter AR immunostaining cells in females (Co vs. Ca). Exogenous prolactin did not cause changes in immunostaining when administered to uncastrated animals, and only reduced epithelial immunostaining when administered for 3 days to castrated animals (Fig. 8a, b).

In males, administration of exogenous prolactin for 3 days in castrated animals decreased AR immunostaining of epithelial cells in comparison with the Co and Ca groups (Fig. 8d, e).

## Discussion

**Fig. 9:**
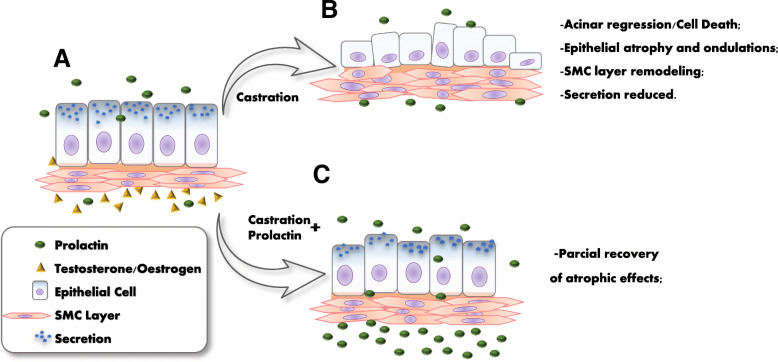
Illustrative scheme of prolactin action in partial recovery from the atrophy prostate caused by castration. A: Control prostate show cubic/prismatic simple epithelium and muscular stroma cell layer (SMC); B: Castrated prostates show acinar regression, epithelium height regresses, the SMC are remodeled to adjust the reduced organ size. C: Castrated with prolactin administration group recovery the atrophic effects of castration, as more voluminous epithelial cells and more organized and compact SMC compared with the castrated animals

Understanding the role of prolactin in the prostate and the way this hormone acts in homeostasis or promoting pathologies of the gland is of immense interest. Although some studies have shown that the administration of prolactin, at the same dosage employed in the present study, produces effects on the rat prostate [40–42], this is the first study to examine the high dose effect of prolactin on the gerbil prostate. In the present study, we have investigated the effects of exposure to high doses of exogenous prolactin in the ventral lobe of the male prostate and in the female prostate of the Mongolian gerbil over 2 treatment periods. The effect of hormone administration was evaluated in intact animals, under the influence of endogenous sex hormones, and in castrated animals, i.e. without the influence of these hormones. The results demonstrated that prolactin changes the morphology of the prostate, acting on both epithelial and stromal cells. The principal morphological alterations are presented in Fig. 9.

We can consider that the dose of prolactin adopted was high, since the serological level of prolactin was around 6.5 ng/mL in the female gerbil in the pre-oestrus phase and 3.7 ng/mL in the male gerbil, according to our measurements; the pathological condition of hyperprolactinemia is defined as circulating PRL levels above the normal range, occurring in conditions other than pregnancy and lactation, when physiological hyperprolactinemia occurs. The major cause of pathological hyperprolactinemia involves tumours of pituitary lactotroph cells (prolactinomas), the main source of PRL in the organism [43].

Exposure to this high dose of prolactin affected the morphology of the gerbil prostate in comparison with the control animals. The animals in the control groups exhibited the general aspect of the gland observed in control females and males similar to that previously described by other authors [11, 15, 44–46] for the prostate of rodents of the same species as those used in this study (*Meriones unguiculatus*).

The morphological effects of castration were also very similar to those already described. The hormone ablation caused by castration resulted in a prostate gland in a process of atrophy previously observed [47–50]. As the epithelium regresses, the stromal cells and extracellular matrix are remodelled to adjust to the reduced organ size [18]. The reduced amount of glycoprotein secretion within the lumens added to a decrease in the nuclear area, the perimeter of the secretory epithelial cells being directly related to reduced synthetic activity of these cells. The accompanying decreases in prostate weight, epithelium height and sml thickness are other indications of the marked process of acinar regression caused by hormonal suppression [51, 52]. The morphometric data showed an increase in epithelial height and sml thickness in the male Ca group. During the atrophy process, the prostatic epithelium regresses and can form curvatures and undulations, which give the impression of having increased in height. In addition, smooth muscle cells become more synthesizing than contractile, and, possibly, produce more constituents of extracellular matrix, which can increase in volume or thickness [18]. However, the other data obtained for this group corroborate the effects established for prostatic regression already described in the literature.

Castration also leads to the appearance of a specific cell phenotype, the spumous cell. This cell type has been observed by our research group with more frequency in male and female gerbil prostates which have undergone the hormonal ablation process due to castration [53–55].

As regards the effect of prolactin administered for a short duration, the prostates of females and males from the PRL 3d group developed several morphological alterations in the epithelium, showing mostly a cribriform aspect to the gland, accompanied by an intense inflammation process. The cribriform pattern of epithelial cell proliferation was also demonstrated in organ culture of the human male prostate after administration of prolactin for 7 days, when epithelial stratification was observed with the formation of microacini [26]. It is known that there is a relationship between the increase of prolactin levels and the increase of prostate diseases in men [21], and also that prolactin is responsible for the development of inflammation in the male gland [56]. The present study demonstrated that similar effects can also occur in the female and male glands of Mongolian gerbils. The morphological atypia observed in these prostates may, in certain cases, develop into prostatic intraepithelial neoplasia (PIN), but not necessarily [57]. A 3-day period of administration is insufficient to be able to characterize such a pre-neoplastic lesion, but it is important to monitor this effect during this treatment.

The period of prolactin exposure influences the actions of this hormone on the prostate, since long-term treatment has shown different results for both male and female gerbils. Treatment with prolactin for 30 days does not result in the same effects as treatment for 3 days. No major foci of inflammation and altered acini were found. The gland, on the other hand, showed a decrease in epithelial volume, revealed in the histological sections by deleting epithelial cell portions within the lumen. Some cell constituents of epithelium had a pyknotic nucleus, surrounded by a clear halo. This process resembles the apoptosis type described by Rosa-Ribeiro et al. [58] for the rat prostate, as the phenomenon of desquamation, a collective epithelial cell deletion particular to androgen deprivation. Further studies with the use of molecular markers for these apoptotic cells will clarify whether it is the same process. A study performed by Herrera-Covarrubias et al. [57] in rats showed that long exposure to exogenous prolactin, from 4 weeks, leads to histological alterations in the prostate that may be considered as precancerous, even in individuals with low levels of androgens. In the present study, which evaluated exposure to prolactin for up to 30 days, the histological changes indicated a more acute effect of prolactin action (3 days) and subsequent structural recovery through cell discard mechanisms. Further studies involving longer-term prolactin exposure may clarify whether the prostate gland develops well-established pre-neoplastic lesions, as occurs in the rat gland.

The presence of ciliated cells in the female prostate is worthy of note. Ciliated cells are seen with some frequency in the prostate epithelium of normal adult female gerbils; however, its role in the physiology of this gland is not well understood. In the present study, ciliated cells were found more often in female prostates from the PRL groups (3d and 30d), to the extent that, in the PRL 3d group, these cells appeared “in groups”. The presence of this cell type is probably related to an abnormal differentiation of basal cells of prostatic epithelium, influenced by a hormonal imbalance [17, 55]. In female prostates treated with androgen and anti-oestrogen hormones, pre-neoplastic and neoplastic alterations appeared in acini which had ciliated cells, so the appearance of these cells can indicate in advance the installation of malignant or pre-malignant lesions [17].

The administration of prolactin promoted the recovery of some regressive aspects of the gland already after 3 days of administration in castrated animals. There was an increase in prostatic weight and nuclear area and perimeter, indicating a tendency to secretory activity of returning epithelial cells. In females there was also an increase in epithelial height and in sml thickness. Although the acini had regressed, the administration of prolactin after castration appears to have mitigated their atrophic effects, without, however, causing the appearance of significant lesions. Some studies have shown that prolactin acts as a growth factor for prostate tissue with an important role in the survival of prostate cells after castration [20, 59–62]. In addition, it increases prostate weight, stimulates DNA and RNA synthesis in all lobes of the gland [63], and increases the levels of zinc and citrate in the lateral lobe (reviewed in Rui and Purvis [64], and in Costello and Franklin [20], all of which effects can occur independently of the presence of androgens.

The location of prolactin and its receptor, in the gerbil prostate, was studied by immunocytochemistry, using an anti-PRL antibody or anti-PRLR antibody, respectively. PRLR undergoes changes in its transmembrane domain after being activated by PRL and can then act as a key regulator of many biological processes, such as growth and metabolism [65]. These receptors are mainly located in the apical region of the secretory epithelium cells. In an immunocytochemistry reaction for PRLR, the nucleus of the cells remained unmarked and low immunostaining was observed in the stromal cells [61]. This same pattern was observed in the gerbil female and male prostates in the present study.

Exogenous prolactin administration in the prostate of non-castrated and castrated animals increased the immunostaining of PRLR and PRL in both sexes, especially after 30 days of administration. In high concentrations, prolactin can saturate the receptor and hinders further receptor dimerization, serving to explain the frequently observed bell-shaped, dose-dependent curves [28]. The present study demonstrated that the dose used was not yet sufficient to reach the maximum binding threshold between hormone and receptor, so that the tissue still remained responsive to increased circulating prolactin.

Although castration did not cause a change in the immunostaining of PRLR, sexual hormonal ablation decreased PRL immunostaining in female and male prostatic tissue alike. In the female this decrease in immuno-marking for PRL is related to decreased serum prolactin levels. The steroid hormones, particularly oestrogen, stimulate the release of prolactin by lactotrophs of the pituitary gland, and the suppression of these hormones, caused by castration, interrupts the stimulation of pituitary prolactin release [24]. We also observed a decrease in pituitary weight associated with castration. In rats, the weight of the pituitary gland increases with increasing body weight of the animal [66], and it is known that female hormones related to reproduction have an influence on pituitary gland volume [67]; it is also known that oestrogens influence the expression of the PRL gene in the pituitary gland and can result in pituitary cell hyperplasia [68].

However, in the male, serum prolactin levels increased after castration. The literature shows conflicting data about prolactin levels in orchiectomized males; some studies have shown an increase [69] and others a reduction [40, 70].

Exogenous exposure to prolactin decreases serum prolactin levels in castrated animals. These data are consistent with those found in the work of Constantino et al. [40], which also observed a decrease in serum prolactin levels in castrated males treated for 10 days with subcutaneous injections of prolactin, at the same doses as those used in the present study. The authors suggested the occurrence of a short loop feedback, where prolactin itself acts in the brain to stimulate the production of dopamine and thereby inhibit its own secretion. Our data also indicate the same phenomenon in the case of gerbil prostates. In intact males, the administration of exogenous prolactin increased PRL serum levels; probably the presence of testosterone maintained high production of endogenous prolactin which, added to the injected prolactin, increased the serum level of the hormone.

In animals that presented lesions, the prolactin receptor did not show increased expression in the affected acini; however, these acini showed high AR immunostaining in males. Androgens regulate the growth and proliferation of prostatic cells. Tumour development results from a multi-step process that initially leads to the formation of low- to high-grade prostatic intraepithelial neoplasia, which is primarily controlled by androgens [71].

The administration of prolactin did not cause changes in prostate AR in uncastrated females but increased them in uncastrated males. It is well established that, in the male prostate, prolactin increases levels of androgen receptors [72]. Gómes and co-workers [73] showed an increase in the number of AR-positive cells after treatment with prolactin in uncastrated rats. Circulating prolactin is also detected in males, although it is present at lower levels than in females. In the male prostate, androgens are related to the differentiation of the secretory epithelium, and growth and regulation of secretory activity [74]. These functions are also related to androgens in the female gland [10]; however, the female body is more controlled by oestrogens and progesterone, which are present in substantially higher levels than testosterone. This may explain why androgen receptors were not influenced by the manipulation of prolactin levels in female prostate tissue.

In castrated males, the administration of prolactin for 3 days decreased AR immunostaining in epithelial and stromal cells. Prolactin acts directly on the prostate in synergy with androgens, stimulating its activity and increasing tissue sensitivity to androgenic action [75]. There is an increase in binding testosterone after incubation of the human prostate with exogenous prolactin [76]. In addition, the increase in circulating levels of prolactin is related to the pathogenesis of prostatic tumours (reviewed in Costello and Franklin [20]). The present study demonstrated the synergistic action between prolactin and androgenic hormones in the ventral prostate of the Mongolian gerbil, showing the dependence of prolactin on the presence of testosterone to promote tissue-specific effects.

The immunostaining of oestrogen receptors ERα and ERβ decreased in the female prostate after castration, whereas AR did not change; these data are consistent with the results previously published by our research group for the gerbil female prostate [52]. In males, there was an increase in ERα expression after castration. Oestrogens are produced from androgens by the action of aromatase [39]. Castration reduces the levels of circulating androgens and oestrogens in the body. This hormonal fall may have led to increased levels of ERα in the tissue in order to increase sensitivity to oestrogen. ERβ expression did not change after castration. The androgenic peak occurring during puberty coincides with a decrease in ERα expression in the prostate, indicating that androgens can suppress the action of these receptors on the tissue [77]. In contrast, androgens increase ERβ expression in the prostate of rodents, whereas oestrogens themselves cannot self-regulate this receptor [78, 79].

In females under normal hormonal conditions, exogenous prolactin administration did not alter ERα expression in prostate tissue, and decreased ERβ only in stromal cells. However, in castrated females, prolactin administration increased ERα and ERβ immunostaining in epithelial and stromal cells. It is well established in the literature that oestrogens exert a strong influence on prolactin levels, stimulating prolactin secretion by the pituitary gland lactotrophs [24]. The present study showed that prolactin, in contrast, influences the oestrogen receptor levels of the female prostate in conditions of low estradiol serum levels, making the tissue more responsive to this hormone. PRL appears to be a key up-regulator of the ER in many reproductive tissues, such as the ovarian corpus luteum [80, 81], the mammary gland and the decidua [82], increasing mRNA and protein levels of both ER subtypes.

In the male, the scenario was the opposite: prolactin administration increased ERα and ERβ expression in the prostate of intact animals but did not alter the expression of ERα in the prostate of castrated animals. An androgen-dependent relationship of prolactin was observed in the male organism, where serum levels of testosterone are much higher and dominate the other steroid hormones and the actions they control. A comparative study between males and females of gerbil evaluated ERα and ERβ expression in the prostate and demonstrated that testosterone reduced ERα expression in the female prostate, while it was necessary for the expression of ERβ in the male gland [34].

## Conclusions

The data presented demonstrate that prolactin and its receptor are expressed in the female and male prostate gland of the Mongolian gerbil and influence its maintenance and homeostasis. The administration of exogenous prolactin to intact animals promotes the appearance of important histological alterations in the gland after just 3 days of administration, as well as a subsequent cellular discarding process that apparently recovers the morphological aspects of the tissue, after 30 days of administration. Prolactin has also been involved in the recovery of the gland after atrophy caused by castration. A relationship of dependence between prolactin and testosterone in the male prostate has been demonstrated, whereas, in the female gland, prolactin establishes a greater performance with the oestrogens, taking into account the differences between the hormonal profiles of the sexes.

## Data Availability

This paper is part of the Thesis presented by M Zanatelli to the Institute of Biology, UNICAMP, in partial fulfilment of the requirement for a PhD in Cell Biology. The original text has public access in the repositories of that University.

## Abbreviations

AR: Androgen receptor
BSA: Bovine serum albumin
Ca: Castrated group
CaP3: Castrated - Prolactin 3 days group
CaP30: Castrated; Prolactin 30 days group
Co: Control group
DAB: Diaminobenzidine
ERα: Estrogen receptor alpha
ERβ: Estrogen receptor beta
H&E: Hematoxylin–eosin
P3: Prolactin 3 days group
P30: Prolactin 30 days group
PAS: Periodic Acid and Schiff
PINCs: Cribiform intraepithelial neoplasms
PINs: Intraepithelial neoplasms
PRL: Prolactin
PRLR: Prolactin receptors
RIA: Radioimmunoassay
